# Supplementary material for: Parameterization of Biomechanical Variables through Inertial Measurement Units (IMUs) in Occasional Healthy Runners
Source: Sensors (Basel). 2024 Mar 29;24(7):2191. doi: 10.3390/s24072191 (PMC11014260; doi:10.3390/s24072191)
Supplement: Supplementary file 1 [file sensors-24-02191-s001.zip › Supplementary Data S1. Flow Chart of Parameterization.pdf]

Pre-measurement

A. Explanation and signature of the informed consent
B. Completion of Self-reported questionaries
C. Length measurement of lower limbs

↓

Placement

Set-up 4 shimmers:

- 2 shins
- 2 lower back

↓

Warm-up

A. Articular movements of lower limbs and lower back

B. 15 quickly squats

↓

Measurement

Running 6´on treadmill at preferred speed

- 30″ of recording data in the first half of the 6th minute
+
- 30 first steps recorded in the 6th minute
+
- 1 first step recorded in the 6th minute
