# Supplementary material for: Parameterization of Biomechanical Variables through Inertial Measurement Units (IMUs) in Occasional Healthy Runners
Source: Sensors (Basel). 2024 Mar 29;24(7):2191. doi: 10.3390/s24072191 (PMC11014260; doi:10.3390/s24072191)
Supplement: Supplementary file 1 [file sensors-24-02191-s001.zip › Supplementary Data S2. Table of Variables.pdf]

FLOW CHART OF VARIABLES GROUPED BY UNIT OF MEASUREMENT

Measurement Unit	Variable	Axis	Accelerometry (Acc) or Gyroscopic (Giro)	IMU
**N/g; g**	1. GRF<br>2. PSD Low<br>3. PSD Mid<br>4. SA<br>5. PPA	1. Y<br>2. r= Y+Z<br>3. r= Y+Z<br>4. r= Y+Z<br>5. Y	1. Acc<br>2. Acc<br>3. Acc<br>4. Acc<br>5. Acc	1. Left and right tibias<br>2. Sacrum (S1)<br>3. Lumbar (L1)<br>4. Sacrum (S1) + Lumbar (L1)<br>5. Left and right tibias
**Seconds**	1. ST<br>2. FT<br>3. GCT<br>4. DF	1. Y<br>2. Y<br>3. Y<br>4. Y	1. Acc and Giro<br>2. Acc and Giro<br>3. Acc and Giro<br>4. Acc and Giro	1. Left Tibia<br>2. Left Tibia<br>3. Left Tibia<br>4. Left Tibia
**Steps**	1. SR	1. Y	1. Acc and Giro	1. Left and right Tibias
**Centimetres**	1. vCOMd	1. Y	1. Acc	1. Sacrum (S1)
**Degrees**	1. ROM	1. Z+Y+X	1. Acc, Giro and Mag	1. Left and right Tibias

N/g: Newton/g force; IMU: Inertial Measurement Unit; r: resultant; Y: craniocaudal axis; X: mediolateral axis; Z: anteroposterior axis; GRF: ground reaction force; PSD Low: power Spectral density of the low back; PSD Mid: power spectral density of the middle back (L1); SA: shock attenuation; PPA: peak positive acceleration; ST: step time; FT: flight time; GCT: ground contact time; DF: duty factor; SR: step rate; vCOMd: vertical center of mass displacement; Mag: magnetometer.
