# Supplementary material for: Parameterization of Biomechanical Variables through Inertial Measurement Units (IMUs) in Occasional Healthy Runners
Source: Sensors (Basel). 2024 Mar 29;24(7):2191. doi: 10.3390/s24072191 (PMC11014260; doi:10.3390/s24072191)
Supplement: Supplementary file 1 [file sensors-24-02191-s001.zip › Supplementary Data S3. Correlations Matrix.pdf]

CORRELATION MATRIX OF 30 SECONDS

	BMI	MASS	PREFERED SPEED	STEP RATE	FLIGHT TIME	GCT	STRIDE TIME	DUTY FACTOR	COM	GRF	GRF BW	MEAN PSD LOW	MEAN PSD MID	PEAK PSD LOW	PEAK PSD MID	SA	PPA	ROM
**BMI**	1.000	**.886***	.268	-.053	-.047	.111	.050	**.488***	-.021	**.895***	**.701***	.355	.039	.330	.046	-.034	.125	.200
**MASS**		1.000	.263	-.097	-.151	.139	.085	**.494***	.133	**.966***	**.711***	.391	-.010	.332	-.018	-.131	.198	.231
**PREFERED SPEED**			1.000	**.515***	**-.551***	**-.448***	**-.493***	.050	.175	.374	**.661***	**.424***	.355	**.433***	.399	.189	.014	-.098
**STEP RATE**				1.000	**-.633***	**-.969***	**-.996***	-.388	-.316	-.011	.235	**.437***	.251	**.473***	.318	.054	-.326	-.316
**FLIGHT TIME**					1.000	**.508***	**.613***	-.215	.226	-.189	-.387	-.263	.012	-.310	-.030	.189	-.098	.039
**GCT**						1.000	**.982***	**.516***	.307	.071	-.171	**-.453***	-.301	**-.490***	-.359	-.066	.334	.369
**STRIDE TIME**							1.000	.406	.320	.006	-.225	**-.443***	-.269	**-.481***	-.333	-.056	.328	.320
**DUTY FACTOR**								1.000	-.041	**.444***	.329	-.067	-.167	-.077	-.132	-.075	.319	.359
**COM**									1.000	.109	.053	.298	.281	.259	.213	.108	-.188	-.041
**GRF**										1.000	**.837***	.374	-.034	.308	-.041	-.160	.126	.184
**GRF BW**											1.000	**.467***	.078	**.424***	.083	-.179	-.021	-.018
**MEAN PSD LOW**												1.000	**.571***	**.977***	**.572***	.045	-.281	-.369
**MEAN PSD MID**													1.000	**.627***	**.985***	**.750***	-.351	**-.467***
**PEAK PSD LOW**														1.000	**.628***	.112	-.300	-.412
**PEAK PSD MID**															1.000	**.770***	-.364	**-.501***
**SA**																1.000	-.177	-.309
**PPA**																	1.000	.418
**ROM**																		1.000

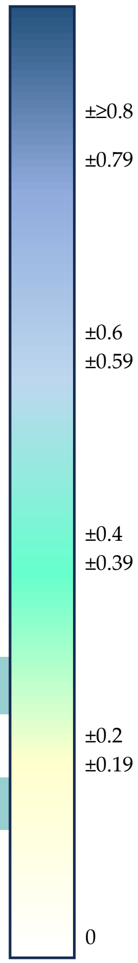

It highlighted in colour only significant results (*)

CORRELATION MATRIX OF 30 STEPS

	BMI	MASS	PREFERED SPEED	STEP RATE	FLIGHT TIME	GCT	STRIDE TIME	DUTY FACTOR	COM	GRF	GRF BW	MEAN PSD LOW	MEAN PSD MID	PEAK PSD LOW	PEAK PSD MID	SA	PPA	ROM
BMI	1.000	.886*	.268	-.043	-.242	.088	.039	.399	.045	.895*	.679*	.355	.053	.357	.068	-.125	.230	.171
MASS		1.000	.263	-.030	-.322	.115	.068	.372	.103	.966*	.690*	.391	.003	.365	.007	-.206	.339	.202
PREFERED SPEED			1.000	.472*	-.223	-.471*	-.530*	.051	.128	.374	.648*	.424*	.367	.462*	.407	.058	.228	-.072
STEP RATE				1.000	-.170	-.958*	-.953*	-.385	-.488*	.028	.248	.480*	.275	.494*	.305	-.104	-.239	-.308
FLIGHT TIME					1.000	.045	.173	-.676*	.312	-.335	-.429*	.018	.292	-.021	.214	.395	-.459*	-.173
GCT						1.000	.982*	.493*	.506*	.045	-.217	-.476*	-.334	-.474*	-.356	.078	.322	.355
STRIDE TIME							1.000	.368	.516*	-.008	-.282	-.449*	-.280	-.462*	-.316	.142	.287	.303
DUTY FACTOR								1.000	.092	.333	.267	-.164	-.222	-.133	-.130	-.178	.423*	.380
COM									1.000	.076	-.046	.153	.188	.160	.129	.298	.045	.090
GRF										1.000	.816*	.374	-.023	.343	-.021	-.252	.289	.159
GRF BW											1.000	.485*	.077	.470*	.089	-.379	.185	-.022
MEAN PSD LOW												1.000	.586*	.963*	.554*	-.064	-.189	-.391
MEAN PSD MID													1.000	.581*	.983*	.649*	-.243	-.447*
PEAK PSD LOW														1.000	.553*	-.030	-.169	-.357
PEAK PSD MID															1.000	.609*	-.199	-.404
SA																1.000	-.034	-255
PPA																	1.000	.500*
ROM																		1.000

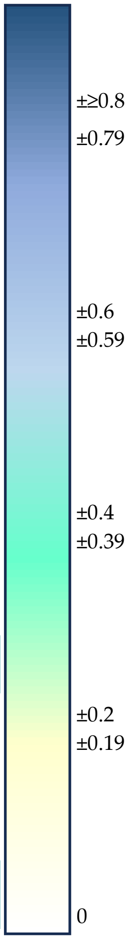

It highlighted in colour only significant results (*)

CORRELATION MATRIX OF 1 STEP

	BMI	MASS	PREFERED SPEED	FLIGHT TIME	GCT	STRIDE TIME	DUTY FACTOR	COM	GRF	GRF BW	MEAN PSD LOW	MEAN PSD MID	PEAK PSD LOW	PEAK PSD MID	SA	PPA	ROM
**BMI**	1.000	**.886***	.268	-.138	.192	-.156	.195	.040	**.912***	**.697***	.365	.048	.318	.051	-.018	.088	.091
**MASS**		1.000	.263	.002	-.024	-.075	.037	.107	**.953***	**.712***	.398	-.003	.321	-.009	-.127	.190	.152
**PREFERED SPEED**			1.000	-.321	-.133	-.403	.286	.153	.393	**.600***	**.442***	.363	**.461***	.410	-.093	.144	-.061
**FLIGHT TIME**				1.000	-.167	**.900***	**-.978***	.099	-.063	-.158	-.231	-.112	-.299	-.116	.251	**.703***	-.005
**GCT**					1.000	.096	.299	.213	.119	.181	-.381	-.355	-.363	-.380	-.106	.022	**.469***
**STRIDE TIME**						1.000	**-.827***	.340	-.121	-.269	-.352	-.168	-.416	-.158	.272	**.591***	.120
**DUTY FACTOR**							1.000	-.030	.104	.174	.200	.060	.255	.066	-.260	**-.648***	.117
**COM**								1.000	.106	-.036	.182	.213	.135	.230	.187	.007	.138
**GRF**									1.000	**.852***	.364	-.027	.303	-.025	-.159	.173	.143
**GRF BW**										1.000	.377	-.002	.350	.003	-.364	.199	.089
**MEAN PSD LOW**											1.000	**.592***	**.980***	**.616***	-.098	-.189	-.360
**MEAN PSD MID**												1.000	**.634***	**.986***	**.577***	-.164	-.415
**PEAK PSD LOW**													1.000	**.661***	-.063	-.260	**-.425***
**PEAK PSD MID**														1.000	**.534***	-.174	**-.470***
**SA**															1.000	.081	-.155
**PPA**																1.000	.315
**ROM**																	1.000

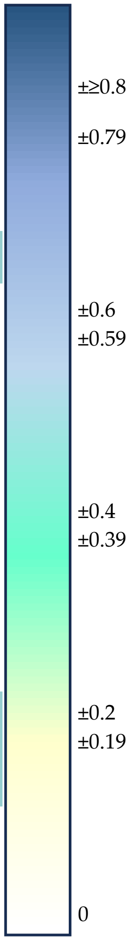

It highlighted in colour only significant results (*)
