# Supplementary material for: Parameterization of Biomechanical Variables through Inertial Measurement Units (IMUs) in Occasional Healthy Runners
Source: Sensors (Basel). 2024 Mar 29;24(7):2191. doi: 10.3390/s24072191 (PMC11014260; doi:10.3390/s24072191)
Supplement: Supplementary file 1 [file sensors-24-02191-s001.zip › Supplementary Data S4. Pairwise comparation.pdf]

PAIRWISE COMPARATION. BONFERRONI TEST

Dependent Variable	(I) WINDOW	(J) WINDOW	Mean Difference (I-J)	Desv. Error	Sig.	Interval of Confidence at 95% Inferior limit	Upper limit
ST	30S	30P	-.00009964860	.02523311469	1.000	-.0621626156	.0619633184
		1P	-.03587132642	.02523311469	.480	-.0979342935	.0261916406
	30P	30S	.00009964860	.02523311469	1.000	-.0619633184	.0621626156
		1P	-.03577167782	.02523311469	.484	-.0978346449	.0262912892
	1P	30S	.03587132642	.02523311469	.480	-.0261916406	.0979342935
		30P	.03577167782	.02523311469	.484	-.0262912892	.0978346449
GCT	30S	30P	-.00381922689	.01781289795	1.000	-.0476315470	.0399930932
		1P	-.00978915215	.01781289795	1.000	-.0536014723	.0340231680
	30P	30S	.00381922689	.01781289795	1.000	-.0399930932	.0476315470
		1P	-.00596992526	.01781289795	1.000	-.0497822454	.0378423949
	1P	30S	.00978915215	.01781289795	1.000	-.0340231680	.0536014723
		30P	.00596992526	.01781289795	1.000	-.0378423949	.0497822454
FT	30S	30P	.00371957829	.01768398502	1.000	-.0397756696	.0472148262
		1P	-.02608217428	.01768398502	.436	-.0695774222	.0174130736
	30P	30S	-.00371957829	.01768398502	1.000	-.0472148262	.0397756696
		1P	-.02980175256	.01768398502	.291	-.0732970005	.0136934954
	1P	30S	.02608217428	.01768398502	.436	-.0174130736	.0695774222
		30P	.02980175256	.01768398502	.291	-.0136934954	.0732970005
DUTY FACTOR	30S	30P	-.00432876825	.01722972515	1.000	-.0467067258	.0380491893
		1P	.01249602598	.01722972515	1.000	-.0298819316	.0548739835
	30P	30S	.00432876825	.01722972515	1.000	-.0380491893	.0467067258
		1P	.01682479423	.01722972515	.998	-.0255531633	.0592027518
	1P	30S	-.01249602598	.01722972515	1.000	-.0548739835	.0298819316
		30P	-.01682479423	.01722972515	.998	-.0592027518	.0255531633
GRF	30S	30P	-3.73697744610	211.2805145458	1.000	-523.3991661386	515.9252112464
		1P	9.60104950217	211.2805145458	1.000	-510.0611391903	529.2632381947
	30P	30S	3.73697744610	211.2805145458	1.000	-515.9252112464	523.3991661386
		1P	13.33802694827	211.2805145458	1.000	-506.3241617442	533.0002156408
	1P	30S	-9.60104950217	211.2805145458	1.000	-529.2632381947	510.0611391903
		30P	-13.33802694827	211.2805145458	1.000	-533.0002156408	506.3241617442
GRF BW	30S	30P	-.00509228091	.09076775095	1.000	-.2283431956	.2181586337
		1P	.01168779682	.09076775095	1.000	-.2115631178	.2349387115
	30P	30S	.00509228091	.09076775095	1.000	-.2181586337	.2283431956
		1P	.01678007773	.09076775095	1.000	-.2064708369	.2400309924
	1P	30S	-.01168779682	.09076775095	1.000	-.2349387115	.2115631178
		30P	-.01678007773	.09076775095	1.000	-.2400309924	.2064708369
PEAK PSD MID	30S	30P	-.00069328433	.00183899582	1.000	-.0052164492	.0038298805
		1P	-.00045904957	.00183899582	1.000	-.0049822144	.0040641153
	30P	30S	.00069328433	.00183899582	1.000	-.0038298805	.0052164492

		1P	.00023423475	.00183899582	1.000	-.0042889301	.0047573996
	1P	30S	.00045904957	.00183899582	1.000	-.0040641153	.0049822144
		30P	-.00023423475	.00183899582	1.000	-.0047573996	.0042889301
PEAK PSD LOW	30S	30P	-.00014755222	.00085102956	1.000	-.0022407310	.0019456265
		1P	-.00016173276	.00085102956	1.000	-.0022549115	.0019314460
	30P	30S	.00014755222	.00085102956	1.000	-.0019456265	.0022407310
		1P	-.00001418055	.00085102956	1.000	-.0021073593	.0020789982
	1P	30S	.00016173276	.00085102956	1.000	-.0019314460	.0022549115
		30P	.00001418055	.00085102956	1.000	-.0020789982	.0021073593
MEAN PSD MID	30S	30P	-.00001643733	.00076363235	1.000	-.0018946553	.0018617806
		1P	-.00002048480	.00076363235	1.000	-.0018987028	.0018577332
	30P	30S	.00001643733	.00076363235	1.000	-.0018617806	.0018946553
		1P	-.00000404747	.00076363235	1.000	-.0018822654	.0018741705
	1P	30S	.00002048480	.00076363235	1.000	-.0018577332	.0018987028
		30P	.00000404747	.00076363235	1.000	-.0018741705	.0018822654
MEAN PSD LOW	30S	30P	.00000680070	.00044962124	1.000	-.0010990805	.0011126820
		1P	.00000802176	.00044962124	1.000	-.0010978595	.0011139030
	30P	30S	-.00000680070	.00044962124	1.000	-.0011126820	.0010990805
		1P	.00000122106	.00044962124	1.000	-.0011046602	.0011071023
	1P	30S	-.00000802176	.00044962124	1.000	-.0011139030	.0010978595
		30P	-.00000122106	.00044962124	1.000	-.0011071023	.0011046602
SA	30S	30P	-.18478154805	1.49200139168	1.000	-3.8544844092	3.4849213131
		1P	-.29333783647	1.49200139168	1.000	-3.9630406976	3.3763650247
	30P	30S	.18478154805	1.49200139168	1.000	-3.4849213131	3.8544844092
		1P	-.10855628841	1.49200139168	1.000	-3.7782591495	3.5611465727
	1P	30S	.29333783647	1.49200139168	1.000	-3.3763650247	3.9630406976
		30P	.10855628841	1.49200139168	1.000	-3.5611465727	3.7782591495
COM	30S	30P	.00115507255	.00503356371	1.000	-.0112254006	.0135355457
		1P	.00095173855	.00503356371	1.000	-.0114287346	.0133322117
	30P	30S	-.00115507255	.00503356371	1.000	-.0135355457	.0112254006
		1P	-.00020333400	.00503356371	1.000	-.0125838071	.0121771391
	1P	30S	-.00095173855	.00503356371	1.000	-.0133322117	.0114287346
		30P	.00020333400	.00503356371	1.000	-.0121771391	.0125838071
PPA	30S	30P	-.62077205012	2.18510177444	1.000	-5.9952135743	4.7536694740
		1P	-.61560613564	2.18510177444	1.000	-5.9900476598	4.7588353885
	30P	30S	.62077205012	2.18510177444	1.000	-4.7536694740	5.9952135743
		1P	.00516591448	2.18510177444	1.000	-5.3692756097	5.3796074386
	1P	30S	.61560613564	2.18510177444	1.000	-4.7588353885	5.9900476598
		30P	-.00516591448	2.18510177444	1.000	-5.3796074386	5.3692756097
